# 3D models for cancer-microbe interaction studies *via* pattern screening: a focus on lung and breast tumoroids

**DOI:** 10.3389/fimmu.2026.1984436

**Published:** 2026-09-16

**Authors:** Martina Mandarano, Alessia Sulla, Gianluca Vascelli, Giorgia Renga, Claudia Floridi, Cristina Pelliccia, Angelo Sidoni, Vasileios Oikonomou, Teresa Zelante

**Affiliations:** 1 Section of Anatomic Pathology and Histology, Department of Medicine and Surgery, University of Perugia, Perugia, Italy; 2 Section of General Pathology, Department of Medicine and Surgery, University of Perugia, Perugia, Italy

**Keywords:** breast cancer, dectin-1, lung adenocarcinoma, microenvironment, organoid, pattern recognition receptors, tumoroid

## Introduction

The complexity and cell heterogeneity of the tumour microenvironment have been extensively delineated in recent years. In particular, the roles of the intratumour microbiome and mycobiome in cancer pathogenesis are increasingly recognised (1, 2). For instance, β-glucan, the cell wall component of the fungus *Aspergillus sydowii*, promotes lung adenocarcinoma (ADC) progression by modulating interleukin (IL)-1β signalling and the accumulation of immunosuppressive regulatory T cells (3). In renal carcinoma, the intratumoural mycobiota signature and the intratumoural mycobiota-related gene expression signature strongly predicted prognosis and immunotherapy outcomes. In this study, *Aspergillus tanneri* was identified as a potential key fungal species affecting cancer prognosis by inducing T cell exhaustion (4). Interestingly, breast cancer has been described as extremely rich in microbiome niches compared to other tumours (5).

All these studies highlight the importance of investigating the expression and function of pattern recognition receptors (PRRs) on cancer cells, which serve as a critical bridge amongst cancer cells, the microbial environment, and the modulation of innate and adaptive immunity.

PRRs bridge innate and adaptive immunity by recognising conserved molecular patterns on pathogens and signals from apoptotic or damaged cells. Based on domain homology, PRRs are classified into five families: Toll-like receptors (TLRs), nucleotide-binding oligomerisation domain (NOD)-like receptors (NLRs), retinoic acid-inducible gene-I (RIG-I)-like receptors (RLRs), C-type lectin receptors (CLRs) and absent in melanoma-2 (AIM2)-like receptors (ALRs) (6). To recognise both extracellular and intracellular threats, PRRs are located on the cell surface and within endosomal compartments. PRRs ligands are broadly classified as microbial-associated molecular patterns (MAMPs), pathogen-associated molecular patterns (PAMPs) and damage-associated molecular patterns (DAMPs). MAMPs and PAMPs include highly conserved structures that are essential for the growth and proliferation of microorganisms (lipopolysaccharides, proteins, nucleic acids), whereas DAMPs are products of host cell metabolism and death (uric acid, bile acid salts) (7). PRR-ligand interactions induce cytokine, chemokine, and growth factor secretion, promoting inflammation and adaptive immune responses. Tumour-derived debris and other products are recognised as DAMPs by PRR-expressing innate immune cells, triggering key responses such as antigen presentation to CD8^+^ T cells and subsequent cytotoxic activity against cancer cells (8). However, PRRs are also expressed by different tumour cells, including colon, lung, breast and gastric cancer cells (7). In this context, PRR activation mainly promotes tumour progression (9).

Tumoroids represent an optimal three-dimensional system for modelling human tumours *in vitro* (10). To date, lung ADC tumoroids have been used to perform tumour gene profiling and evaluate tumour drug susceptibility, morphology, and biomarker expression patterns at the air-liquid interface (11). Genomic, epigenomic, proteomic, and histological analyses reliably demonstrated preservation of tumour-specific alterations and lineage features. Together, these studies establish lung tumoroids as realistic patient avatars, providing a robust foundation for functional precision drug testing. Similarly, breast cancer (BC) tumoroids have been used for diagnosis and high-throughput drug testing (12).

Here, we assemble further insights into the dynamic links between PRRs, microbiota, and cancer, suggesting how 3D models may become a personalised strategy to investigate *ex vivo* how individual cancer cells may interact with microbial patterns and how this interaction may affect cancer development, progression, or therapy response. In addition, tumoroids can be used to monitor the levels of DAMPs or PAMPs along with PRR expression. The level of innate activation determines the ability to recognise bacterial, viral, or fungal components, eventually contributing to tumour progression by modifying the microenvironment.

## From the microbiome to PRRs: the inner microbial side of the lung and breast tumour microenvironment

Lung cancer is the leading cause of cancer-related deaths worldwide (13). Non-small-cell lung cancer (NSCLC) accounts for ~80% of cases (13), with ADC being the most common histotype. ADC is characterised by glandular differentiation and typically displays a mixture of histological patterns that serve as prognostic markers. The 5^th^ World Health Organisation (WHO) classification of thoracic tumours (14) recognises five ADC patterns: lepidic, acinar, papillary, micropapillary, and solid (listed in order of declining prognosis). Immune checkpoint inhibitors, particularly those targeting the programmed death ligand 1, have expanded treatment options, and advanced-stage, non-oncogene addicted tumours with a tumour proportion score ≥50% are eligible for first-line immunotherapy (15). However, many ADCs are characterised by inadequate anti-tumour T lymphocyte activation, highlighting the need for tumour microenvironment (TME) remodelling to improve the antitumor response.

The intratumour microbiome has recently emerged as a key determinant of lung ADC onset, progression, and prognosis (3, 16). By activating innate receptors, the intratumour microbiome strongly affects the TME (17). Moreover, specific microbes may trigger chronic inflammation, which also affects tumour metastasis. A recent study characterised the intratumour microbiome in lung ADC (18). Liu et al. demonstrated that the intratumour microbiome of ADC is enriched in the fungus *Aspergillus sydowii* (3). *A. sydowii* promoted tumour progression by increasing IL-1β levels and recruiting myeloid-derived cells, thereby driving the accumulation of regulatory T cells within the TME. This was the first study to document the presence of *Aspergillus* within ADC tumour tissues and demonstrate that its cell wall component β-glucan could mobilise immunosuppressive cells within the tumour by activating the Dectin-1/CARD9 signalling pathway.

Meanwhile, Su et al. demonstrated that the most abundant bacterial genera found in lung ADC were *Pseudomonas*, *Streptococcus*, *Mycobacterium*, *Neisseria*, and *Mesorhizobium.* Patients with late-stage lung ADC exhibited a more complex intratumour microbiome than those with early-stage disease, providing evidence of microbiome differentiation (19). Patients with advanced lung adenocarcinoma had significantly higher levels of the bacterial genera *Pseudoalteromonas*, *Luteibacter*, *Caldicellulosiruptor*, and *Serratia* than patients with less advanced disease (19). More recently, multiomics analysis revealed that early-stage lung ADC was associated with intratumour lung dysbiosis. *Methyloversatilis discipulorum* abundance was significantly positively correlated with GOLM1 expression at both the transcript and protein levels. Finally, machine learning revealed that six bacterial markers successfully distinguished patients with early-stage lung ADC from healthy control subjects (20). A recent study used a mouse model of lung ADC to show that, mechanistically, intratumour dysbiosis was linked to chronic inflammation and tumour progression. Stone and colleagues inoculated an animal cancer model driven by mutant K-ras and Tp53 with *Acidovorax temperans*, which is enriched in lung tumours. They found that *A. temperans* accelerated tumour development and increased tumour burden by promoting the infiltration of proinflammatory cells into the tumour, causing a global shift away from IL-1β signalling and enriching T helper (Th)17 cells in the TME. This TME remodelling was associated with a gene expression programme predictive of poor survival in human lung ADC (21). Thus, chronic exposure to certain types of bacteria may promote tumour growth by modulating inflammation.

Several studies identified the expression of PRRs on both cancer cells and innate myeloid cells in the TME, although results are still controversial and their function is unclear (22, 23). Expression analysis revealed that the levels of TLR1/2/3/4/5/7/8 were decreased in lung ADC, whilst the levels of TLR6/9/10 were increased. Lung ADC patients with low expression of TLR1/2/3/5/8 and high expression of TLR9 had poorer overall survival (24). Another study revealed that TLR3 expression is detectable in both early-stage lung cancer cells and myeloid cells. Very interestingly, the expression pattern in non-myeloid and myeloid cells has an opposite prognostic significance and highlights the value of measuring the levels of this receptor, both in tumour cells and infiltrating immune cells (25). The stimulation of PRRs is also under study, as for example demonstrated via the use of imprime, a soluble β-glucan ligand of Dectin-1, which did not impact the outcome of advanced-stage patients with lung cancer. Further investigation is needed to understand how therapeutic PAMPs mount a strong innate immune response against cancer (22).

BC remains the most frequently diagnosed cancer in women and the most common malignancy worldwide. It accounted for roughly 2.3 million new cases estimated in 2020, and its global incidence continues to rise (26). Pathological classification distinguishes carcinoma *in situ* and invasive carcinoma based on whether neoplastic cells arising from the ductal or lobular breast epithelium breach the basement membrane and invade the surrounding stroma (27). The intratumour breast microbiome has been investigated by several researchers in recent years. An interesting study demonstrated that Gal-GalNAc levels increase along the progression of human breast cancer and that *F. nucleatum* becomes overabundant in human BC samples, particularly in those with high Gal-GalNAc signals. Importantly, colonisation of tumours by *F. nucleatum* accelerates breast cancer progression and metastatic development *via* T cell reduction in the TME. Breast tumour exacerbation by *F. nucleatum* in mice can be counteracted by treatment with metronidazole (28). More recently, it has been shown that intratumour microbiota in human and murine breast is characterised by a high presence of intracellular bacteria such as *Enterococcus* and *Streptococcus*, playing crucial roles in tumour metastasis by modulating cellular cytoskeleton and cell viability upon mechanical stress (29).

High TLR expression has been linked to BC aggressiveness and therapeutic resistance (30). For example, TLR4 promotes BC cell invasiveness and is associated with local tumour progression and lymph node metastasis (31). BC cells are highly responsive to TLR5 activation; indeed, it has been shown that tumour proliferation is inhibited when flagellin, a TLR5 conventional ligand, is added in mouse models (32). In addition, the combination of TLR4 and TLR5 stimulation triggers inflammation, breast acini and DNA damage in a 3D model of breast cancer (33). In addition, Dectin-1 expression has been observed in the breast TME and appears to correlate with poorer patient survival (34). However, Dectin-1 and other fungal recognition receptors of the same family (Dectin-2, Dectin-3 and Mincle) have been shown to play a dual pro- or anti-tumour role, where results are still controversial (35).

## Generation of human tumoroids: pattern screening and PRR stimulation in personalised medicine

3D cancer cell cultures are expected to advance personalised medicine in cancer. There is evidence that lung ADC can be modelled using tumoroids derived from biopsies, excised tumours, or induced pluripotent stem cells. To overcome the limited air exposure of conventional cultures, lung ADC tumoroids can be co-cultured with healthy lung fibroblasts at the air-liquid interface, facilitating drug screening by providing direct access to the apical and basal surfaces of cancer cells (11, 36). More recently, lung tumoroids have been generated from a rare subtype of lung ADC, invasive mucinous adenocarcinoma (37). Interestingly, these tumoroids produced more MUC5AC than other cultured tumour cell lines, and their inoculation into immunodeficient mice generated tumours resembling the primary patient tumours (37).

Organoid cultures have recently emerged as a valuable tool for translational BC research, revolutionising the study of healthy and diseased organ biology. BC organoid morphology appears to accurately reflect tumour histology, with ductal carcinomas forming solid organoids and lobular carcinomas forming discohesive structures (38). Moreover, BC organoids have been successfully generated and used to establish biobanks that accurately reflect the heterogeneity of the tumours from which they are derived, including histopathology, hormone receptor expression, and copy number variations (39). Breast tumoroids can also be engrafted into mice to generate preclinical *in vivo* models (29, 37). More recently, bioengineered organoids (40) generated using multiplex CRISPR-Cas9 gene-editing technology have been developed to enhance cytotoxic T-cell-mediated tumour clearance and reduce the recruitment of tumour-infiltrating neutrophils (40). Thus, tumoroids have been used as a screening tool for cancer- associated antigens and T cell cytotoxicity assays (41). Similarly, tumoroids may be used as a platform for PAMP, MAMP, DAMP screening and PRR activation upon delineation of the tumour microbiome (**Figure 1**).

**Figure 1:**
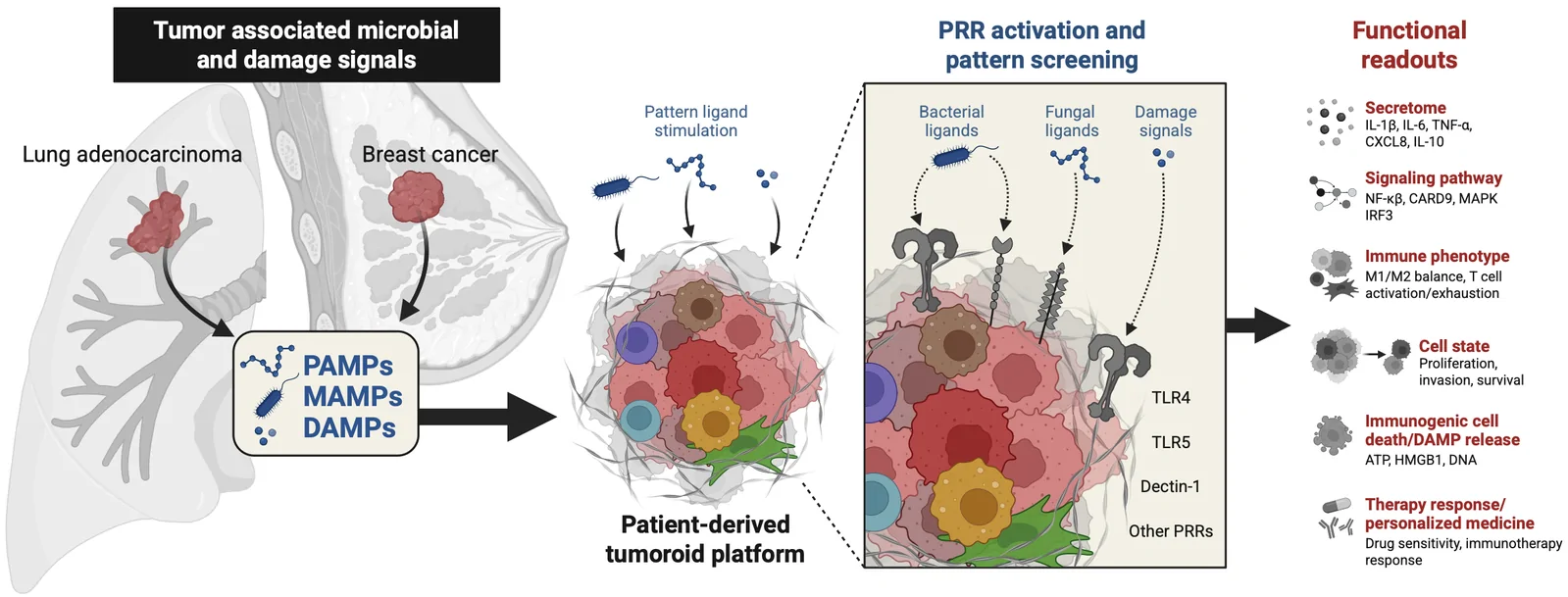
Pattern screening on tumours: advances in PRR understanding. Lung or breast tumoroids may be useful to build a platform for PAMP, MAMP, DAMP screening and PRR activation upon delineation of the tumour microbiome. Several responses and functional readouts may help to better understand the TME. Created in BioRender. Oikonomou, V. (2026) https://BioRender.com/0nqxo9u.

Indeed, a novel aspect that has emerged in recent years is that tumours harbour a microbiome and mycobiome that shape the TME (42). In line with this notion, tumoroids may represent a platform to study how PRRs influence cancer pathogenesis through the recognition of MAMPS, PAMPs or DAMPS. For example, TLR4 and Dectin-1 recognise the bacterial and fungal PAMPs lipopolysaccharide (LPS) and β-glucan, respectively. These interactions are particularly relevant because both bacteria and fungi are components of the lung intratumour microbiome (3).

Importantly, high Dectin-1 expression in tumour tissues is an independent predictor of poor prognosis in several types of cancer (3, 43). Similarly, the crucial roles of intracellular bacteria and LPS in solid tumours highlight TLR4 as a promising prognostic marker in ADC (5). Ultimately, tumoroids may represent useful tools for understanding how cancer cell adaptation occurs with the microbiome and the whole microenvironment.

## Discussion

In conclusion, tumoroids are useful tools for investigating interactions amongst the intratumour microbiome, the TME of the parental tumour, and antitumour immunity. Lung and breast tumoroids closely mimic the histological features of the patient-derived tissue and enable the identification of PRRs that recognise microbial components linked to cancer progression. By supporting in-depth characterisation of the TME and the intratumour microbiome, tumoroids may accelerate the discovery of new therapeutic targets and enhance TME-remodelling immunomodulatory strategies.

The increasing heterogeneity of the disease, widespread resistance to available drugs, and frequent metastatic recurrence after treatment remain major challenges in cancer research. These challenges highlight the urgent need for representative *in vitro* models that accurately reflect disease complexity.
